# The effects of dietary fasting on physical balance among healthy young women

**DOI:** 10.1186/1475-2891-9-18

**Published:** 2010-04-13

**Authors:** Shanthi Johnson, Krista Leck

**Affiliations:** 1 Faculty of Kinesiology and Health Studies, University of Regina, 3737 Wascana Parkway, Regina, Saskatchewan S4S 0A2, Canada; 2 Formerly, School of Nutrition & Dietetics, Acadia University, 12 University Avenue, Wolfville, Nova Scotia, B4P 2R6, Canada

## Abstract

**Background:**

The study examined the effects of dietary fasting on physical balance among young healthy women.

**Methods:**

This study undertaken involving 22 young healthy women (age = 22 ± 1.5) using a within subject counterbalanced 2-week crossover study design. Participants were asked to refrain from consuming any food or beverage for 12 hours prior to the fasting trial and to maintain their regular diet for the non-fasting trial. Measures included: a background questionnaire, 24-hour dietary recall, and functional reach and timed single-limb stances.

**Results:**

Fasting resulted in significant declines in functional reach (p < 0.01), and ability to balance in a single limb stance with eyes open, on both the dominant and non-dominant legs (p < 0.01 and p < 0.01, respectively), and with eyes closed on the dominant leg (p < 0.01).

**Conclusions:**

The findings have implications for athletic performance in younger individuals as well as emphasizing the need for health education for young women to avoid skipping meals.

## Background

The importance of balance can be seen in every aspect of daily living: walking up stairs, bending over to tie shoes or standing still. While the human body must be able to adapt to its environment and movements in order to maintain this balance, the loss or impairment of balance influences quality of living, decreases athletic performance, and can lead to injury [1,2]. Balance refers to an individual's ability to maintain the body in mechanical equilibrium and relies on specific postural mechanisms and sensory inputs (e.g., visual, somatosensory, vestibular) the purpose of which is to promote an appropriate alignment of body posture to maintain an upright stance against the force of gravity [3,4]. The two main aspects of balance that have been critically assessed are static and dynamic balance. Static balance is balance while the body is at rest, while dynamic balance refers to the ability to maintain balance during movement [1,5-7].

Balance is a complex and multifaceted fitness parameter and is assessed in a number of different ways. In young and old individuals, balance is generally assessed using functional performance tasks that evaluate everyday living skills (e.g., obstacle courses, timed up and go tests or functional reach tests) as well as more challenging situations (e.g., modifying a sensory input and the use of firm and compliant surfaces). Measures such as functional reach have also been used to quantify dynamic balance in all age groups [8]. Similarly, static balance can be assessed using a timed single limb stance procedure (e.g., eyes closed or open or a maximum timed duration), or by using a force plate to measure and quantify postural sway [9-12]. With minimal disturbances in balance, the ankle strategy is used to regain postural stability. However, as the difficulty increases, the ankle is no longer able to maintain control and the body shifts to using other postural mechanisms such as the hip strategy and/or upper limb movement [6]. Failure to recover balance through a combination of these mechanisms will ultimately result in a fall.

Studies suggest that there are many conditions or situations which can disrupt the control of balance. Age-related physiological changes, the presence of injury, disease, and medication, can alter the ability to maintain balance. Additionally, some research has reported sex differences in the ability to maintain balance [13,14]. This difference has been attributed to womens' lower centre of gravity, which in turn improves their ability to balance [13,14]. Research has also shown that physically active individuals have an increased balancing ability over sedentary individuals of a similar stature [15]. However, over the short-term, fatiguing exercise has been shown to reduce balance [16]. While physical activity and nutrition are important parts of overall health, limited research, to date, has examined the specific relationship between nutrition and balance. The few studies that do exist have used balance tests as a secondary measurement within an overall study of nutrition and performance among elite male athletes, and show that a decrease in dietary intake negatively affects one's ability to maintain balance [17-20].

Fasting is a state of nutritional imbalance, which is generally characterized by an absence or interruption of caloric intake over a period of time [21]. Physiologically, the body shifts into a state of early fasting, also known as a post-absorptive state, 3 to 12 hours after eating. After 12 hours of fasting, the post-absorptive state shifts to that of a fasting state, which is characterized by hypoglycemia or low blood sugar levels and the associated increase in gluconeogenic activity, with amino acids being the primary source of substrate [21-23]. Although nutrition imbalance has not been identified as a direct risk factor related to falls, the symptoms of hypoglycemia include a lack of coordination, staggering gait, fatigue, disorientation, dizziness, and vertigo. These symptoms are well-known risk factors for impaired balance and falls in the elderly population [24-26]. It is not uncommon for younger and older individuals to practice partial fasting [27]. People may skip meals and not eat for extended periods of time due to a self-imposed weight loss strategy, delayed eating due to the administration of medication or medical testing (e.g., fasting blood tests), or because of adherence to religious practices. The interruption of caloric intake places additional stress on the body, and could undermine the body's ability to perform daily living tasks.

The potential implication of fasting on functional capacity has not been well examined. Studies investigating the effects of fasting on functional performance, as determined by various fitness indicators such as endurance and balance, have used a wide variety of fast durations, ranging from 14 hours to 3.5 days [19,20]. A limited number of studies show that fasting contributes to decreased endurance capacity and reaction time, as well as reduced static and dynamic balance performance, along with increased heart rate and blood lactate levels during exercise, all of which are detrimental to performance [17,18]. Studies have shown that the ability to maintain balance is affected after fatiguing exercise [16]. Researchers have found that compensatory mechanisms exist to help maintain balance and these mechanisms are more active after fatiguing exercise [1,19,20]. The majority of these studies, however, have involved functionally elite men such as athletes or soldiers [17-20]. None of the studies involved individuals from the general population or women. As indicated earlier sex differences in the ability to maintain balance have been reported [13,14]. In addition, young women are more likely to skip meals and not eat for extended periods of time due to a self-imposed weight loss strategy. Studies have examined the role of skipping breakfast on cognition but not physical function in general and balance in particular with young women [27]. Thus, the purpose of this study was to examine the effects of dietary fasting on physical balance among young women.

## Methods

### Participant selection and study design

A sample of 22 Caucasian women was recruited for this study. Given the lack of research in this area involving young healthy women, power analysis was not performed. Participants were included only if they had no health conditions that could be worsened by fasting or that could affect their ability to balance (diabetes, recent/chronic head injuries and/or lower extremity disabilities, low blood pressure, vestibular and/or inner ear problems). Participant recruitment was completed through advertisement in the University bulletin after the study design and protocol were approved by the authors' institutional Research Ethics Board. The participants provided signed informed consent prior to participation.

The present study adopted a within subject counterbalanced crossover design, given the many advantages inherent in this design--all subjects served as their own controls and therefore reduced possible error variance, while reducing the needed sample size. Each participant was tested under fasting and non-fasting conditions, with half of the participants randomly assigned to start under the non-fasted condition and the remaining half completed the fasted trial first. The conditions were reversed during the second trial and the two trials were separated by a two week duration time span to avoid learning effects. All participants completed the same set of tests at each trial, in the same order. Standardized testing procedures and equipment were used throughout the study. The testing took place in the investigator's research laboratory. Snack bars and juice boxes were available for the participants to consume after study testing. No other incentives were provided to the participants.

### Fasting and non-fasting protocol

Participants were provided with the fasting/non-fasting protocol prior to each test period. Thus, it was not possible to blind study participants to the testing condition. All laboratory tests took place between 9 and 11 am in order for all participants to avoid time of day variations. For the fasting trial, participants were asked to refrain from consuming food or beverages for a minimum of 12 hours prior to testing. Specifically, all participants were instructed to consume an evening meal before 8 pm prior to the day of testing and to refrain from any food or beverages until the time of testing in the laboratory. Fasting protocol was planned to mimic the scenario of skipping breakfast which is common among young women, or overnight fasting necessary for certain blood tests (e.g., fasting blood glucose). For non-fasting tests, participants were instructed to maintain normal eating habits on both the day prior to and the day of testing. In both test conditions, participants were asked to refrain from strenuous physical activity to minimize the role of carry-over effects such as fatigue, muscle damage, or physiological potentiation. Prior to each testing period, a pre-trial checklist was completed to provide information on each participant's past 24 hours of activity (e.g., timing of last meal, exercise performed, and injuries/health episodes since the previous contact).

### Measures

The measures included: a background questionnaire, nutritional intake, balance tests, and a pre-trial checklist.

#### Background questionnaire

This questionnaire was used to elicit information related to the participants' demographic characteristics (age, education, ethnicity, employment, and financial status) and health status (perceived health, tobacco use, and physical activity level). The questionnaire was completed by the participants once, prior to the first testing period.

#### Nutritional intake

A 24-hour food recall was used to collect data on average caloric intake of the participants. The recalls were conducted by the student investigator trained in the protocol, using a standard form and adopting strategies to avoid recall bias by asking the participants to record food intake for 24 hours prior to the food recall interview [28]. Recalls were collected once during the study period and at mid-point between the two trials.

### Physical testing

Physical testing included: 1) dynamic balance and 2) static balance. These measures were completed twice, once in the fasted condition and again in a non-fasted condition.

#### Dynamic balance

The functional reach (FR) test was used to assess dynamic balance [8]. For this test, the difference in centimetres between the participant's standing arm length and maximal forward reach was recorded. While standing with feet flat on the floor, the standing arm length was measured using a metric measuring tape fixed to the wall. Participants were then asked to reach as far forward as possible, without taking a step, without their heels leaving the ground or without losing balance. Three trials were performed with the farthest reach identified as the final score.

#### Static balance

The single limb stance was used to assess static balance [1]. This test can be performed in various time durations (e.g., 10, 25, 45 seconds or maximum time to exhaustion or termination) as well as with and without the use of a force platform. In the present study, the test was conducted on the gym floor and the maximum duration the participant could maintain a single limb stance was recorded in seconds on both the dominant and non-dominant legs, as well as in eyes open and eyes closed conditions [29], to avoid ceiling effect, given the involvement of healthy young women in this study. The dominant leg was determined by assessing which foot took the first step when participants were asked to initiate gait. Participants stood on the dominant leg, raising their non-dominant leg to a 90 degree angle at the hip and the knee joints while keeping their hands down by their sides. In this study, the test was terminated if the participant voluntarily asked to stop for any reason (e.g., sore leg), if the knee dropped below 90 degrees, if one placed the foot on the floor, or began to use arms to maintain balance. The reasons for the termination of the test were recorded for each of the four static balance testing conditions. The same set of termination criteria were used in both the fasting and non-fasting trials. Trials were conducted using the same protocol for both legs and under both conditions--eyes open and eyes closed. A practice trial was provided to each participant to ensure proper form was used during the actual trial. One test trial was completed for each condition to prevent fatigue and practice effects.

#### Pre-trial checklist

During each test session, participants completed a pre-trial checklist. This checklist gathered information related to fasting duration, level of physical activity prior to testing, and any change in health since recruitment or first testing.

### Data analysis

Data from the two trials were numerically coded and entered into a Statistical Package for the Social Science database [30] for analyses. Descriptive statistics (means and frequencies) were used to describe the demographic and health characteristics as well as balance measures. Subsequently, paired t-test was conducted to examine the changes in balance performance scores for the two trial conditions. Completed dietary recalls were entered into a dietary analysis program, Food Processor SQL [31], and were used to calculate an average nutrient intake for a 24-hour period. Similarly, foods consumed for breakfast (in the same 24-hour period) were analyzed using Food Processor SQL to calculate the average nutrient intake. Based on the difference of nutrient intake for the whole day versus breakfast, an average "caloric loss" for fasted trials was calculated. The significance level was set at (p ≤ 0.05) for all statistical procedures.

## Results

A total of 22 female, Caucasian university students participated in this study without any attrition between trials. Descriptive statistics on the demographic characteristics and health status are presented in Table 1. The age of study participants ranged from 18 to 23 years with a mean of 21.8 years (SD = 1.5). Of the study participants, 95.4% rated their perceived health status as excellent to good and all the participants were non-smokers. Over 90% of the study participants consumed alcohol on a regular basis. The majority of the participants reported performing regular physical activity (90.9%) with a range of four to six days a week (63.6%) for a duration of 30 minutes or longer (90.9%) each time. The perceived health and physical activity status was self report and no definitions were provided. None of the participants reported any injuries or medical conditions that could affect their balance.

**Table 1:** Descriptive statistics on the demographic and health characteristics of the participants

Characteristics	n = 22
Age in Years (mean ± SD)	21.77 ± 1.5
Number of Years in University (mean)	3.5 ± 1.3
Ethnicity (%)	
Caucasian	100
Employment Status (%)	
Part-time	63.6
Do not work during school year	36.4
Financial Status (%)	
Easily meets needs	40.9
Just meets needs	40.9
Barely meets needs	18.2
Perceived Health Status (%)	
Excellent to good	95.4
Fair to poor	4.5
Non-smoker (%)	100
Perceived Regular Physical Activity (% yes)	90.9

Data from the pre-trial checklist showed that a minimum 12-hour fast was followed by the participants during the fasted trial, with a mean fast of 15 hours. As expected, there was a statistically significant difference in the time of last meal for the fasted and non-fasted trials (p = 0.011). The average daily caloric intake was 2062 kcals with 15.2% of these calories coming from protein, 60.2% from carbohydrates and 28% from fat. When analyzing the breakfast meal individually, the average total calories from breakfast alone was 465.8 kcals or 22.6% of the day's total calories. The breakfast meal consisted primarily of carbohydrate calories (74.6%) and interruption of the consumption of breakfast contributed to an average caloric deficit of 465.8 kcals during the fasted trial.

Table 2 presents the mean scores for each of the balance measures assessed, based on the trial condition (fasted or non-fasted). For the single limb stance test, there was a decrease in duration (seconds) of the participants' ability to maintain balance as the level of difficulty increased, from eyes open, dominant leg to eyes closed, non-dominant leg in both fasted and non-fasted trials, albeit the fasted trial had consistently lower scores compared to non-fasted trial. Similarly, for the functional reach test, the participants' score was lower in the fasted trial compared to non-fasted trial. Paired comparison t-test showed that fasting resulted in statistically significant declines in functional reach (p < 0.01), the ability to balance in a single limb stance with eyes open, on both the dominant and non-dominant leg (p < 0.01 for both) and with eyes closed trial on the dominant leg (p < 0.01) among healthy young adults. No statistically significant change was observed for the static balance test in the eyes closed condition on the non-dominant leg (p = 0.13).

**Table 2:** Comparison of scores of physical measures between non-fasted and fasted trials

Trial	Non-fasted	Fasted	p-value
Measures	n = 22	n = 22	
Dynamic Balance (cm)			
Functional Reach (mean ± SD)	42.1 ± 4.5	38.8 ± 4.3	0.000
Static Balance (sec)			
Eyes Open:			
Dominant Leg (mean ± SD)	101.0 ± 46.4	80.0 ± 36.4	0.011
Non-Dominant Leg (mean ± SD)	93.3 ± 39.6	75.4 ± 38.4	0.006
Eyes Closed:			
Dominant Leg (mean ± SD)	21.5 ± 18.0	13.1 ± 9.8	0.002
Non-Dominant Leg (mean ± SD)	17.8 ± 15.9	14.7 ± 12.3	0.131

Along with the decline in balance performance between trials, an increased postural response (e.g., stumble, use of arms, knee lowered) was recorded as the reason for test termination as the degree of difficulty progressed in the single limb stance (from eyes open dominant leg to eyes closed non-dominant leg) and from the non-fasted to fasted trial (see Table 3). Specifically, in non-fasted conditions, the frequency of stumbling as the reason for termination increased with the increase in the level of difficulty in the four trials (from eyes open, dominant leg trial to the eyes closed non-dominant leg trial). However, under fasted conditions, participants showed an increase in postural responses, mainly stumbling, leading to termination in both the eyes open and closed conditions in the dominant leg but the trend was not true for non-dominant leg. In each of the trials, those in the fasted condition reported higher frequency of stumble compared to those in non-fasted condition except for the trial with the highest level of difficulty (eyes closed, non-dominant leg) in which the scores in seconds were similar (as shown in Table 2).

**Table 3:** Reasons for Termination of Single Limb Balance Trial (Percentage reporting Postural Response)

Trial	Non-fasted	Fasted
Test Condition	n = 22	n = 22
Eyes open, Dominant leg (%)		
Stumble	22.7	27.3
Sore Ankle/Leg/Hip	59.0	45.4
Knee Lowered	18.2	9.0
Use of Arms	--	18.2
Eyes open, Non-dominant leg (%)		
Stumble	13.6	36.4
Sore Ankle/Leg/Hip	63.5	36.4
Knee Lowered	9.0	13.6
Use of Arms	13.6	13.6
Eyes closed, Dominant leg (%)		
Stumble	68.2	86.4
Sore Ankle/Leg/Hip	13.5	--
Knee Lowered	--	--
Use of Arms	18.2	13.6
Eyes closed, Non-dominant leg (%)		
Stumble	77.3	77.3
Sore Ankle/Leg/Hip	4.5	4,5
Knee Lowered	4.5	--
Use of Arms	13.6	18.2

## Discussion

The present study provided insights into the relationship between dietary fasting on static and dynamic balance. Specifically, in a sample of physically active and healthy young women, dietary fasting of a 12-hour period affected the individuals' ability to maintain dynamic and static balance as measured using the functional reach and single limb stance tests, respectively. As indicated earlier, there is a paucity of research examining this issue among young women who are more likely to skip meals and not eat for extended periods of time to lose weight. No other study was found in the literature which examined static and dynamic balance in fasting condition in women, although decline in balance has been reported in studies which involved functionally elite men such as athletes or soldiers [17-20].

In addition to the decreased duration of balance ability between fasted and non-fasted trials, increased postural control mechanism responses were noted which resulted in the termination of the test. In the present study, at the easier stages of the single limb stance test (eyes open), the majority of the participants stopped due to sore leg. However, as the difficulty increased (eyes closed) more of the body was required to maintain postural control resulting in more stumbling and use of arms to aid balance. Under fasting conditions, during both the eyes open and eyes closed trials, the majority of the stances were terminated due to a stumble. This is consistent with previous studies which suggest that individuals use various postural strategies for the maintenance of balance after fatiguing exercise [1,19,20]. The fasting condition placed additional stress on the body's ability to balance and therefore, required a larger postural response in order to maintain balance.

In the present study, a minimum fasting duration of 12 hours was adopted. Although this is slightly lower than the range of 14 hours to 3.5 days of fasting duration reported in existing studies, it is similar to the standard fasting protocols required for certain blood tests and sufficiently long for the body to switch from a post-absorptive state into a fasting state [17-21]. By using a fast duration that mimicked a skipped breakfast scenario or fasting blood test, the study protocol reflected a common fasting situation in the general population. The results can therefore be applied to everyday situations, instead of what happens under extreme fasting situations (e.g., 3.5 days of fasting). In the present study, it was estimated that fasting could have contributed to a 465.8 kcals deficit or approximately 25% of daily intake when compared to the non-fasted condition. Although there are no comparable data, studies have used a standard breakfast of 595 kcals for male participants [17]. Further research is needed to examine the physiological mechanisms underlying the relationship between fasting and balance including the role of energy regulating hormones (insulin, leptin, adiponectin, ghrelin) as well as the resulting metabolic and biochemical changes (e.g., hypoglycemia). Association between nutrition and falls should be examined further in the population of older adults [26].

While the results of the present study have furthered our understanding of the relationship between dietary fasting and its effect on physical balance among healthy, young women, it is not without limitations. The study recruited participants through a non-random sampling approach. As such, self selection bias would be inherent in the sampling approach. Also, with the lack of research in this area, it was not possible to calculate an appropriate sample size. However, based on the changes observed in the present study in the functional reach test, which is an indicator of dynamic balance, it can be estimated that 20 participants are required for each group to study whether there is a gender difference in the observed effects of fasting. Additionally, it was not possible to gather dietary data in fasting and non-fasting states. Future studies should calculate the actual caloric deficit between the two conditions.

The present study also had several strengths. The use of a within subject counterbalanced crossover design was a major strength of this study. This set-up allowed many advantages; all participants performed the tests in fasted and non-fasted conditions, served as their own controls and therefore reduced possible error variance, while reducing the needed sample size. This is the first study to date that has examined the relationship between dietary fasting and physical balance as its primary focus within the general population, with the use of a common fasting duration (e.g., skipping breakfast). Standardized testing procedures and protocols were used to minimize variance errors and ensure reproducible data.

The findings raise issues to be addressed in future research. For example, while a small decline in balance may not translate into difficulties in performing activities of daily living in younger individuals or have serious clinical significance in the day to day functioning given the higher levels of functional threshold in young individuals, these small changes in balance may affect athletic performance if the young women are also actively competing in sport in which balance is integral to performance (gymnastics, skating). Also, changes in balance could limit elderly individuals from performing basic activities as well as predispose them to high risk for falls. Future studies are needed to test the sport performance and fall risk prepositions. Falling is a complex problem influenced by a multiplicity of risk factors, yet the inability to maintain balance (i.e., postural instability) is one of the best predictors of falls among the elderly individuals [24,32]. Given the high incidence of falls and the debilitating consequences such as hip fracture among the older population [25], future studies are needed to demonstrate the effects of aging on the relationship between fasting and balance. In these studies, the appropriateness of measure such as a single limb stance with increasingly difficulty conditions (eyes closed) for frail older adults and the related safety concerns should be considered.

## Competing interests

The authors declare that they have no competing interests.

## Authors' contributions

CSJ contributed to the conceptualization and design, as well as to the analysis, interpretation and writing of the article. KRL contributed to the conceptualization and design, data collection and analysis, and writing of the article. Both authors have read and approved the final manuscript.
